# Seed amplification assay results illustrate discrepancy in Parkinson’s disease clinical diagnostic accuracy and error rates

**DOI:** 10.1007/s00415-023-11810-2

**Published:** 2023-08-17

**Authors:** John Stephen Middleton, Hanna Lynn Hovren, Nelson Kha, Manuel Joseph Medina, Karen Ruth MacLeod, Luis Concha-Marambio, Kendal Jay Jensen

**Affiliations:** 1 Clinical Laboratory, Amprion Inc, San Diego, CA USA; 2 Research Laboratory, Amprion Inc, 10355 Science Center Drive, San Diego, CA 92121 USA

**Keywords:** α-Synuclein, Seed amplification, Synucleinopathy, Parkinson’s disease

## Abstract

Parkinson’s disease (PD) may be misdiagnosed due to the clinical overlap between PD and atypical parkinsonism. The utility of α-Synuclein (αSyn) Seed Amplification Assay (SAA) as a diagnostic indicator for PD has been reported in numerous studies, but never when administered as a validated clinical laboratory test. This study compares results from αSyn-SAA validation testing performed using well-characterized cohorts from two biorepositories to better understand the accuracy of PD clinical diagnosis. Blinded cerebrospinal fluid (CSF) specimens from a repository that included cohorts of subjects clinically diagnosed as PD or healthy controls, both with confirmatory dopamine transporter single-photon emission computed tomography (DAT SPECT) imaging, and blinded CSF specimens from a repository that included cohorts of subjects clinically diagnosed as PD or healthy controls based on clinical diagnosis alone, were tested as part of the validation studies for the diagnostic αSyn-SAA test (SYNTap® Biomarker Test). Measured αSyn-SAA test accuracy was 83.9% using clinical diagnosis as comparator, and 93.6% using clinical diagnosis with confirmatory DAT- SPECT imaging as comparator. The statistically significant discordance between accuracy determinations using specimens classified using different diagnostic inclusion criteria indicates that there is some symbiosis between dopamine-weighted imaging and αSyn-SAA results, both of which are associated with higher accuracy compared with the clinical diagnosis alone.

**Supplementary Information:**

The online version contains supplementary material available at 10.1007/s00415-023-11810-2.

## Introduction

Approximately 20% of Parkinson’s disease (PD) may be misdiagnosed due to the clinical overlap between PD and atypical Parkinsonism [1]. Non-PD Parkinsonism disorders confound the diagnosis of PD and may have different pathological mechanisms (e.g., tauopathy, TDP-43 aggregation, etc.), and management [2]. Dopamine transporter single-photon emission computed tomography (DAT-SPECT) identifies deficits in presynaptic domine levels and is useful for demonstrating nigrostriatal degeneration occurring in PD and other diagnoses involving dopamine deficit [3, 4]. DAT-SPECT findings reflect dopaminergic function and do not necessarily identify the exact underlying etiology when a deficit is identified. However, when used for the indication of aiding PD diagnosis in cohorts with suspected PD (high pre-test probability), DAT-SPECT strengthens the accuracy of diagnosis [5]. The Parkinson’s Progressive Markers Initiative (PPMI) study included DAT-SPECT imaging to enhance the accuracy of diagnosis for PD and control cohorts with the rationale that individuals with “scans without DAT deficit are unlikely to have PD.” [5]. An accurate PD biomarker test is therefore expected to have a high correlation to PPMI cohort assignment, since there is high confidence in the accuracy of clinical diagnosis associated with these specimens.

The α-Synuclein (αSyn) Seed Amplification Assay (SAA) is a novel biomarker test that has significant support in the clinical research community. The assay utilizes in vitro propagation of minute amounts of aggregated misfolded αSyn to achieve levels detectable by simple fluorescence measurements of the amyloid-specific dye, Thioflavin T (ThT) [6–9]. αSyn‑SAA reproducibility across laboratories and methodological variations has been previously demonstrated; however, no study has been performed in a CLIA (Clinical Laboratory Improvement Amendments)/CAP (College of American Pathologists) accredited laboratory, and no study has compared cohorts selected with and without diagnostic imaging using SAA as the comparator [10]. To this end, we compared assay results between two cerebrospinal fluid (CSF) repositories using a CLIA/CAP validated αSyn-SAA, and compared test performance to repository inclusion diagnostic criteria.

## Materials and methods

All patient specimens were collected under institutional review board approved protocols and with informed consent.

### Study populations

For the first study, clinical data and frozen CSF from 98 participants were obtained from the Parkinson's Disease Biomarkers Program (PDBP) biorepository [11]. PDBP is a consortium in which participants are assessed longitudinally using standardized sample collection protocols and clinical assessments using Movement Disorder Society or UK Brain Bank criteria [12]. We initially identified case samples from subjects with PD (*n* = 41) and then identified control samples age and sex-matched to case samples to the extent possible (*n* = 57).

**Fig. 1:**
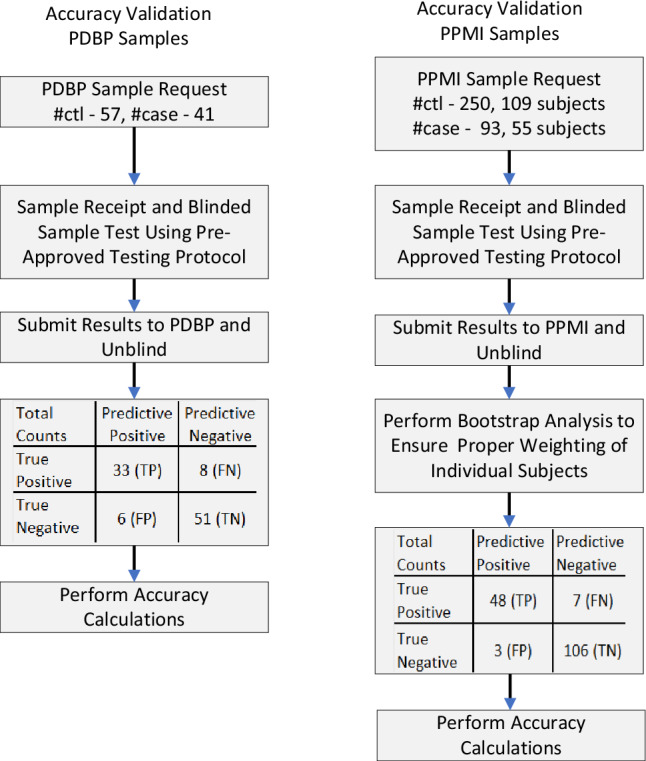
Flow chart of participants included in the study; the truth tables were constructed using αSyn-SAA results (predictive) compared against the repository clinical classifications (presumed true)

For the second study, different specimens from different individuals were obtained from the Parkinson’s Progression Markers Initiative (PPMI) repository [5, 10]. Unlike the PDBP repository, the PPMI enrollees require an abnormal DAT-SPECT test in addition to clinical symptoms to be included in the PD cohort and a normal DAT-SPECT test to be included in the control cohort. A total of 343 blinded samples from the PPMI biorepository were tested. The 343 samples were composed of 250 specimens from 109 individuals classified as controls, and 93 specimens collected from 55 individuals classified as PD. Specimens from participants who were clinically diagnosed with PD but had a normal DAT-SPECT on visual inspection (Scans Without Evidence of Dopaminergic Deficit; SWEDD) were not included in this study [5, 13]. In most instances where multiple samples were tested for one subject, these were collected from different visits. To appropriately weigh the influence of individual subjects on the statistics, results within the subject were randomly selected through computer-generated modeling to determine the most likely accuracy metrics for reporting. (Refer to Online Resource for additional information.) Fig. 1 depicts the study populations’ testing for both cohorts.

### SAA procedure

Briefly, a 2.45 mm diameter borosilicate glass bead (Sigmund Lindner GmbH, cat# 55-02450-89RTS), 160 µL of the reaction mixture, and 40µL of test sample were combined in each test well of a 96-well plate, and the plate was sealed with an optical adhesive film (Applied Biosystems, cat# 4311971). The reaction mixture used throughout the validation studies was similar to formulations described in previous studies [10, 14] and was composed of 100 mM PIPES (MilliporeSigma, cat#80635) pH 6.5, 10 µM ThT (MilliporeSigma, cat#T3516-25G), nuclease-free water (Growcells, cat#NUPW-0500), 500 mM NaCl (Lonza, cat#51202), and 0.3 mg/mL recombinant monomeric human αSyn (Amprion, cat#S2020). Three test wells are used for each patient assessment. A BMG LABTECH FLUOStar Ω Microplate Reader (excitation wavelength, 440 nm; emission wavelength, 490 nm) was used to measure ThT fluorescence in relative fluorescence units (RFU). Following a baseline reading, the assay plate was incubated at 37 °C and subjected to cycles of orbital shaking at 800 rpm for 1 min followed by a pause for 29 min. Fluorescence readings were taken once per day for 7 days with the exception of infrequent extended cases requiring 10 days. Extension occurs when a partial fluorescence signal has evolved near the end of the 7-day read window. Following the final measurement, the maximum relative fluorescence units (RFU) of each well is determined and the median of the 3 wells for each sample is calculated. Samples with median signal values greater than or equal to 25,000 RFU are classified as “Detected” (positive for aggregates of misfolded αSyn), and samples with median signal values < 25,000 RFU are classified as “Not Detected” (negative for aggregates of misfolded αSyn).

### Statistics

The positive predictive value, negative predictive value, sensitivity, specificity, accuracy, and error rate (false results divided by the total number of results) are reported. 95% confidence intervals were calculated using the binomial confidence interval calculator in https://statpages.info/confint.html. Graphs and tables were generated using Microsoft Excel. Statistics were performed using R version 4.1.1 with statistical significance set to 0.05.

## Results

**Fig. 2:**
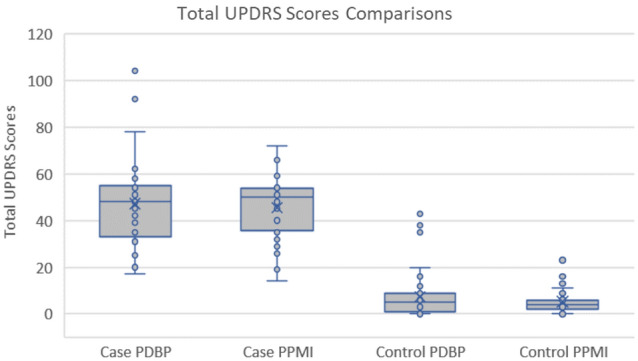
Box and whisker plots of the total UPDRS Scores broken down by repository and case/control cohorts. The median UPDRS scores for the PDBP and the PPMI control and case subjects were 4 and 5 and 48 and 50, respectively. *p* values from robust *T* tests (Yuen) were 0.37 and 0.73, respectively, when comparing control and case scores between repositories

We first looked at potential demographic differences between PPMI and PDBP repositories and found no statistical differences in age, race, or ethnicity apart from race and gender for the control cohort comparison (Table 1). To verify the PDBP and PPMI cohorts’ comparability in terms of symptomology, United Parkinson’s Disease Rating Scale (UPDRS) data were analyzed; there were no statistical differences between repositories (Table 1, Fig. 2).

**Table 1:** Summary of Cohort Parameters. UPDRS comparisons were performed using robust T tests (Yuen), age comparisons were performed using the Kruskal–Wallis Rank Sum Test, and Race Ethnicity Sex used Pearson’s Chi-squared Tests

Parameter	Subject group						*p* values from repository comparisons	
	PPMI repository			PDBP repository				
	Case	Control	Total	Case	Control	Total	Case	Control
Total UPDRS scores non-parametric summary								
Median	50	4	–	48	5	–	0.727	0.371
IQR	18.5	4	–	22	8	–		
Min	14	0	–	17	0	–		
Max	72	25	–	104	43	–		
Demographics comparison								
Age								
Mean	64.0	60.1	61.4	64.3	61.4	62.6	0.932	0.882
Median	64.0	64.0	64.0	63.6	64.0	63.6		
SD	9.0	11.8	11.1	8.0	10.0	9.3		
Min	34.2	31.0	31.0	48.4	39.4	39.4		
Max	77.5	85.1	85.1	81.0	84.4	84.4		
< 60	16 (29.1%)	53 (48.6%)	69 (42.1%)	12 (29.3%)	27 (47.4%)	39 (39.8%)		
> 60	39 (70.9%)	56 (51.4%)	95 (57.9%)	29 (70.7%)	30 (52.6%)	59 (60.2%)		
Race								
Caucasian	52 (94.5%)	99 (90.8%)	151 (92.1%)	37 (90.2%)	47 (82.5%)	84 (85.7%)	0.395	0.041
Black	1 (1.8%)	7 (6.4%)	8 (4.9%)	3 (7.3%)	10 (17.5%)	13 (13.3%)		
Other	2 (3.6%)	3 (2.8%)	5 (3.0%)	1 (2.4%)	0 (0.0%)	1 (1.0%)		
Ethnicity								
Hispanic	2 (3.6%)	2 (1.8%)	4 (2.4%)	1 (1.6%)	2 (3.5%)	3 (2.5%)	1.000	0.879
Not Hispanic	53 (96.4%)	107 (98.2%)	160 (97.6%)	59 (96.7%)	54 (94.7%)	113 (95.8%)		
Unknown	0 (0.0%)	0 (0.0%)	0 (0.0%)	1 (1.6%)	1 (1.8%)	2 (1.7%)		
Sex								
Male	38 (69.1%)	68 (62.4%)	106 (64.6%)	23 (56.1%)	24 (42.1%)	47 (48.0%)	0.274	0.020
Female	17 (30.9%)	41 (37.6%)	58 (35.4%)	18 (43.9%)	33 (57.9%)	51 (52.0%)

An important difference between these repositories is the inclusion criteria, which includes DAT-SPECT in the PPMI cohort. Considering repository diagnosis as the comparator, αSyn-SAA accuracy was higher in the PPMI cohort (93.6%) than in the PDBP cohort (83.9%) (Table 2). It is important to note that the error rate for SAA using the PPMI cohort was 6.4% compared to 16.1% in the PDBP cohort (*p* 0.045) (Table 2). For information, additional validation results, including analytical validation and specimen stability studies, are shown in the Online Resource.

**Table 2:** αSyn-SAA test accuracy summary

Metric	PDBP repository samples # controls—57, # PD—41	PPMI repository samples # controls—109, # PD—55
Classification method	Clinical diagnosis w/out DAT SPECT	Clinical diagnosis with DAT SPECT
Accuracy	85.7% (95% CI = 0.77–0.92)	93.9% (CI = 0.89–0.97)
Sensitivity	80.5% (95% CI = 0.65–0.91)	87.3% (CI = 0.76–0.95)
Specificity	89.5% (95% CI = 0.78–0.96)	97.2% (CI = 0.92–0.99)
Error rate	14.3% (CI = 0.080–0.228)	6.1% (CI = 0.030–0.109)
PPV	84.6% (95% CI = 0.69–0.94)	94.1% (CI = 0.84–0.99)
NPV	86.4% (95% CI = 0.75–0.94)	93.8% (CI = 0.88–0.98)

## Discussion

The αSyn-SAA results reveal discordance in the accuracy of clinical diagnosis between two repositories of samples collected from donors diagnosed with PD based on different criteria, namely clinical diagnosis with and without ancillary imaging/enrichment by DAT SPECT. Discordance was anticipated since clinical diagnosis of PD based on clinical evaluation alone carries a significant misdiagnosis rate of ~ 20% [1, 15] and is known to be enhanced with a demonstration of the presence or absence of degenerative findings by DAT-SPECT [16].

Because of the relation between higher αSyn-SAA accuracy and enrichment by DAT-SPECT in the inclusion criterion, we endorse that diagnoses associated with specimens obtained from the PPMI are close to clinical truth (precision between Parkinson diagnostic rate and Parkinson pathobiology). Enrichment by αSyn-SAA alone might reach similar or higher PD diagnostic accuracy than clinical diagnosis enhanced by DAT-SPECT, since αSyn-SAA reaches high accuracy in the PPMI cohort (~ 94%) and DAT-SPECT enrichment is not 100% diagnostic [17]. Moreover, αSyn-SAA has been shown to be more sensitive in borderline PD cases of the PPMI cohort [14]. Dopaminergic depletion is hypothesized to occur later in the disease process than αSyn misfolding, so it is possible that the positive αSyn-SAA samples with normal DAT-SPECT are at earlier stages of the disease. In theory, the provider may find utility in either of these methods depending on the clinical presentation since both DAT-SPECT and αSyn-SAA studies have shown utility in refining PD etiology by uncovering information from two discrete areas in the PD landscape.

These hypotheses were confirmed in a recently published research study using > 1100 PPMI CSF specimens and representing the largest analysis to date of αSyn-SAA [18]. Similar to the results in the present study using PPMI specimens, this study showed sensitivity for sporadic PD of 93.3%, and specificity for healthy controls of 96.3%. The larger PPMI study also included specimens from SWEDD, prodromal, and non-manifesting genetic carriers. Sensitivity for prodromal PD (hyposmia or REM behavior sleep disorder) was 86%. Importantly, there was evidence that abnormal αSyn aggregation detectable by αSyn-SAA occurs before other detectable clinical or biomarker changes, including DAT-SPECT. In addition, αSyn-SAA provided information about molecular heterogeneity, with αSyn-SAA positivity lower in *LRRK2* PD (67.5%) and higher in *GBA* PD (95.9%) compared with sporadic PD. Overall, the authors conclude that αSyn-SAA classifies people with PD with high accuracy and can play a crucial role to identify pathologically defined subgroups of people with PD and to establish a biomarker-driven definition of the disease. αSyn-SAA technology has been extensively proven in the research environment, and the test has been officially validated per CLIA and CAP guidelines allowing a significant advancement in diagnostic biochemistry that has the capacity to change the diagnostic approach to neurodegenerative diseases. Accurate “rule-in” and “rule-out” of synucleinopathies with a biochemical test promises utility since diagnosis of synucleinopathies can otherwise consume long periods of time and potentially involve unnecessary drug challenges. This validation also serves as a precedent for a new class of clinical testing using protein aggregation science in a CLIA-certified high-complexity laboratory. Such a test will also aid in clinical research trials requiring a validated test platform for subject screening or monitoring.

### Supplementary Information

Below is the link to the electronic supplementary material.

Supplementary file1 (DOCX 184 KB)

## Data Availability

The data that support the findings of this study are available from the corresponding author upon reasonable request.
