# Supplementary material for: Seed amplification assay results illustrate discrepancy in Parkinson’s disease clinical diagnostic accuracy and error rates
Source: J Neurol. 2023 Aug 17;270(12):5813–8. doi: 10.1007/s00415-023-11810-2 (PMC10632284; doi:10.1007/s00415-023-11810-2)
Supplement: Supplementary file 1 — Supplementary file1 (DOCX 184 KB) [file 415_2023_11810_MOESM1_ESM.docx]

**Acta Neuropathologica**

**Seed Amplification Assay Results Illustrate Discrepancy in Parkinson’s Disease Clinical Diagnosis Error Rates**

**Authors:** John Stephen Middleton^1^ BS., Hanna Lynn Hovren^1^ BS., Nelson Kha^1^ BS., Manuel Joseph Medina^1^ BS., Karen Ruth MacLeod^1^ BS., Luis Concha-Marambio^2^ PhD., Kendal Jay Jensen^1^ M.D. PhD.

**Affiliations:** ^1^ – Amprion Inc., Clinical Laboratory, San Diego, CA. ^2^ – Amprion Inc., Research Laboratory, San Diego, CA.

**Corresponding Author Contact Information:** Kendal Jay Jensen, 10355 Science Center Drive, San Diego, CA, 92121. Phone Number: 858-461-6338. Email: [Kjensen@amprionme.com](mailto:Kjensen@amprionme.com)

**SUPPLEMENTAL ONLINE CONTENT**

**eMethods.** PPMI Sampling Analysis Method

**eTable1.** Summary of PPMI Sampling Analysis

**eTable2.** Assay Precision Summary

**eTable3.** Approximate Interferent Concentrations for Specificity Evaluation

**eTable4.** Specificity Testing Results Summary

**eTable5.** CSF Specimen Handling and Stability Study Conditions

**eTable6.** Impact of CSF Collection Fraction on α-Syn SAA Test Result

**eTable7.** Impact of Centrifugation on α-Syn SAA Test Result

**eTable8.** Real-Time Stability of CSF: α-Syn SAA

**eTable9.** Freeze-Thaw Stability of CSF: α-Syn SAA

**eTable10.** Limit of Detection Evaluation Hit Rates (# hits/# total wells) Summary

**eFigure1.** Limit of Detection Hit Rates vs. Dilution Plots

**eMethods.** PPMI Sampling Analysis Method

A simple bootstrap evaluation was performed to properly weigh the influence of individual patients on the statistics. Specifically, using a simple Excel Visual Basic script, 1 result within each unique patient set was randomly selected 10,000 times and statistics were calculated for each of the 10,000 data sets that resulted. The results of this analysis are tabulated in eTable1.

**eTable1.** Summary of PPMI Sampling Analysis (10,000 Trials). The modes of the accuracy metrics are reported.

| **Specificity** | **%** |  | **Sensitivity** | | | **%** | | |  | **Accuracy** | | | | **%** | | |  |  |
| --- | --- | --- | --- | --- | --- | --- | --- | --- | --- | --- | --- | --- | --- | --- | --- | --- | --- | --- |
| **Outcome** | **Prevalence** | **count** | **Outcome** | | | **Prevalence** | | | **count** | **Outcome** | | | | **Prevalence** | | | **count** |  |
| 0.954 | 4.89 | 489 | 0.836 | | 3.39 | | | 339 | | 0.921 | | 1.00 | | | | 100 | | |
| 0.963 | 20.66 | 2066 | 0.855 | | 25.55 | | | 2555 | | 0.927 | | 7.78 | | | | 778 | | |
| 0.972 | 37.62 | 3762 | 0.873 | | 39.32 | | | 3932 | | 0.933 | | 22.46 | | | | 2246 | | |
| 0.982 | 28.44 | 2844 | 0.891 | | 24.65 | | | 2465 | | 0.939 | | 26.25 | | | | 2625 | | |
| 0.991 | 8.38 | 838 | 0.909 | | 7.09 | | | 709 | | 0.945 | | 23.75 | | | | 2375 | | |
|  |  |  |  | |  | | |  | | 0.951 | | 13.57 | | | | 1357 | | |
|  | sum | 10000 |  | | sum | | | 10000 | | 0.957 | | 4.79 | | | | 479 | | |
|  |  |  |  | |  | | |  | | 0.963 | | 0.40 | | | | 40 | | |
|  |  |  |  | |  | | |  | |  | |  | | | |  | | |
|  |  |  |  | |  | | |  | |  | | sum | | | | 10000 | | |
|  |  |  |  | |  | | |  | |  | |  | | | |  | | |
|  | **accuracy** | **specificity** | **sensitivity** | **error rate** | | |  | | | | **PPV** | | **NPV** | |  | | | |
| **correct** | 154 | 106 | 48 | 10 | | |  | | | | 48 | | 106 | |  | | | |
| **total** | 164 | 109 | 55 | 164 | | |  | | | | 51 | | 113 | |  | | | |
|  | 0.939 | 0.972 | 0.873 | 0.061 | | |  | | | | 0.941 | | 0.938 | |  | | | |
|  |  |  |  |  | | |  | | | |  | |  | |  | | | |
| **low** | 0.891 | 0.922 | 0.755 | 0.030 | | |  | | | | 0.838 | | 0.877 | |  | | | |
| **high** | 0.970 | 0.994 | 0.947 | 0.109 | | |  | | | | 0.988 | | 0.975 | |  | | | |
| **95% CI** |  |  |  |  | | |  | | | |  | |  | |  | | | |

**eTable 2**. Assay Precision Summary

| **Sample** | **# Not Detected** | **# Detected** | **% Correct** | **95% Confidence Limits** | | **Protocol Acceptance Criteria (% Correct)** | **Status** |
| --- | --- | --- | --- | --- | --- | --- | --- |
|  |  |  |  | **Low** | **High** |  |  |
| Negative 1 | 23 | 1 | 95.8 | 0.789 | 0.999 | ≥95.0% | pass |
| Negative 2 | 23 | 1 | 95.8 | 0.789 | 0.999 | ≥95.0% | pass |
| Low Positive | 3 | 21 | 87.5 | 0.676 | 0.973 | ≥75.0% | pass |
| Medium Positive | 0 | 24 | 100.0 | 0.858 | 1.000 | ≥90.0% | pass |
| Positive | 1 | 23 | 95.8 | 0.789 | 0.999 | ≥95.0% | pass |

### **eTable 3**. Approximate Interferent Concentrations for Specificity Evaluation

| **Interferent** | **Spiking Source Material** | **Levels** |
| --- | --- | --- |
| Whole blood | Venous blood – no anticoagulant | CSF sample to appear as pink (low) and red (high) |
| Hemoglobin | Interference Test Kit For Assay Validation, Molecular Depot, Catalog # K2010001 | 20 and 100 mg/dL |
| Conjugated Bilirubin | Interference Test Kit For Assay Validation, Molecular Depot, Catalog # K2010001 | 1.4 and 6.6 mg/dL |
| Albumin | Albumin Human 10% solution, Sigma, Catalogue # A6784 | 154 and 769 mg/dL |

**eTable4**. Specificity Testing Results Summary

| **Negative Sample Preparations** | | **Positive Sample Preparations** | | |
| --- | --- | --- | --- | --- |
| Sample | Results | Sample | Results |  |
| Neat Sample | Not Detected | Neat Sample | Detected |  |
|  | Not Detected |  | Detected |  |
| High Albumin Level | Not Detected | High Albumin Level | Detected |  |
|  | Not Detected |  | Detected |  |
| Lower Albumin Level | Not Detected | Lower Albumin Level | Detected |  |
|  | Not Detected |  | Detected |  |
| High Bilirubin Level | Not Detected | High Bilirubin Level | Detected |  |
|  | Not Detected |  | Detected |  |
| Lower Bilirubin Level | Not Detected | Lower Bilirubin Level | Detected |  |
|  | Detected |  | Detected |  |
| Diluted Sample | Not Detected | Diluted Sample | Detected |  |
|  | Not Detected |  | Detected |  |
| High Hemoglobin Level | Not Detected | High Hemoglobin Level | Detected |  |
|  | Not Detected |  | Detected |  |
| Lower Hemoglobin Level | Not Detected | Lower Hemoglobin Level | Detected |  |
|  | Not Detected |  | Detected |  |
| High Whole Blood Level | Not Detected | High Whole Blood Level | Detected |  |
|  | Not Detected |  | Detected |  |
| Lower Whole Blood Level | Not Detected | Lower Whole Blood Level | Detected |  |
|  | Not Detected |  | Detected |  |

## **eTable5**. CSF Specimen Handling and Stability Study Conditions

| **Parameter Being Evaluated** | **Storage Temp.** | **CSF Collection Fraction^1^** | **Freeze/Thaw Cycle^2^** | **Planned Storage Time** |
| --- | --- | --- | --- | --- |
| Collection Fraction | -80 °C | Beginning (1^st^ mL)  Middle (2^nd^-11^th^ mL pool)  End (12^th^ mL) | 1 | 1 Day |
| Post-Collection Centrifugation^3^ | -80 °C | Middle | 1 | 1 Day |
| Real-Time Stability | RT | Middle | 0 | 1, 3, 7, 14 Days |
|  | 2–8 °C | Middle | 0 | 1, 3, 7, 14, 30 Days |
|  | -20 °C | Middle | 1 | 1 Day; 6 Months; 4 Years |
|  | -80 °C | Middle | 1 | 1 Day; 1, 6 Months; 1, 2, 4 Years |
| Freeze/Thaw Stability | -80 °C | Middle | 1, 2, 3 | N/A |

### **Abbreviations**: N/A: not applicable; Temp. = temperature

### ^1^ CSF is collected by lumbar puncture. The beginning collection fraction is the 1^st^ mL, the middle fraction is a pool of the 2^nd^-11^th^ mL, and the end fraction is the 12^th^ mL.

### ^2^ For each freeze/thaw cycle, sample aliquots are frozen by placing in a -80 °C freezer and thawed on the bench top.

### ^3^All other listed conditions use samples that were not subjected to post-collection centrifugation.

**eTable6.** Impact of CSF Collection Fraction on α-Syn SAA Test Result

| **Amprion ID No.** | **Clinical Assign.** | **CSF Collection Fraction** | **Result** |
| --- | --- | --- | --- |
| P1 | Healthy Control | Beginning | Not Detected |
|  |  | Middle | Not Detected |
|  |  | End | Not Detected |
| P2 | Healthy Control | Beginning | Not Detected |
|  |  | Middle | Not Detected |
|  |  | End | Not Detected |
| P3 | Healthy Control | Beginning | Not Detected |
|  |  | Middle | Not Detected |
|  |  | End | Not Detected |
| P4 | PD | Beginning | Detected |
|  |  | Middle | Detected |
|  |  | End | Detected |

**Abbreviations**: Assign. = assignment; ID = identification; PD = Parkinson’s Disease

**eTable7.** Impact of Centrifugation on α-Syn SAA Test Result

| **Amprion ID No.** | **Clinical Assign.** | **Post-Collection Centrifugation?** | **Test Result** |
| --- | --- | --- | --- |
| P1 | Healthy Control | Yes | Not Detected |
|  |  | No | Not Detected |
| P2 | Healthy Control | Yes | Not Detected |
|  |  | No | Not Detected |
| P3 | Healthy Control | Yes | Detected |
|  |  | No | Not Detected |
| P4 | PD | Yes | Detected |
|  |  | No | Detected |

**Abbreviations**: Assign. = assignment; ID = identification; PD = Parkinson’s Disease

**eTable8.** Real-Time Stability of CSF: α-Syn SAA

| Amprion ID No. | Clinical Assign. | Time Point | RT Result | 2–8°C Result | -20°C Result | -80°C Result |
| --- | --- | --- | --- | --- | --- | --- |
| P1 | Healthy Control | Day 1 | Not Detected | Not Detected | Not Detected | Not Detected |
|  |  | Day 3 | Not Detected | Not Detected | -- | -- |
|  |  | Day 7 | Not Detected | Not Detected | -- | -- |
|  |  | Day 14 | Not Detected | Not Detected | -- | -- |
|  |  | 1 Month | -- | Not Detected | -- | Not Detected |
|  |  | 6 Month | -- | -- | Not Detected | Not Detected |
|  |  | 1 Year | -- | -- | -- | Not Detected |
| P2 | Healthy Control | Day 1 | Not Detected | Not Detected | Not Detected | Not Detected |
|  |  | Day 3 | Not Detected | Not Detected | -- | -- |
|  |  | Day 7 | Not Detected | Not Detected | -- | -- |
|  |  | Day 14 | Not Detected | Not Detected | -- | -- |
|  |  | 1 Month | -- | Not Detected | -- | Not Detected |
|  |  | 6 Month | -- | -- | Not Detected | Not Detected |
|  |  | 1 Year | -- | -- | -- | Not Detected |
| P3 | Healthy Control | Day 1 | Not Detected | Not Detected | Not Detected | Not Detected |
|  |  | Day 3 | Not Detected | Not Detected | -- | -- |
|  |  | Day 7 | Not Detected | Not Detected | -- | -- |
|  |  | Day 14 | Not Detected | Detected | -- | -- |
|  |  | 1 Month | -- | Not Detected | -- | Not Detected |
|  |  | 6 Month | -- | -- | Not Detected | Not Detected |
|  |  | 1 Year | -- | -- | -- | Not Detected |
| P4 | PD | Day 1 | Detected | Detected | Detected | Detected |
|  |  | Day 3 | Detected | Detected | -- | -- |
|  |  | Day 7 | Detected | Detected | -- | -- |
|  |  | Day 14 | Detected | Detected | -- | -- |
|  |  | 1 Month | -- | Detected | -- | Detected |
|  |  | 6 Month | -- | -- | Detected | Detected |
|  |  | 1 Year | -- | -- | -- | Detected |

**Abbreviations**: Assign. = assignment; ID = identification; RT = Room Temperature; PD = Parkinson’s Disease; -- = data not available at the time of report preparation

**eTable9.** Freeze-Thaw Stability of CSF: α-Syn SAA

| Amprion ID No. | Clinical Assign. | Freeze/Thaw Cycle^1^ | Result |
| --- | --- | --- | --- |
| P1 | Healthy Control | 0 | Not Detected |
|  |  | 1 | Not Detected |
|  |  | 2 | Not Detected |
|  |  | 3 | Not Detected |
| P2 | Healthy Control | 0 | Not Detected |
|  |  | 1 | Not Detected |
|  |  | 2 | Not Detected |
|  |  | 3 | Not Detected |
| P3 | Healthy Control | 0 | Not Detected |
|  |  | 1 | Not Detected |
|  |  | 2 | Not Detected |
|  |  | 3 | Not Detected |
| P4 | PD | 0 | Detected |
|  |  | 1 | Detected |
|  |  | 2 | Detected |
|  |  | 3 | Detected |

**Abbreviations**: Assign. = assignment; ID = identification; PD = Parkinson’s Disease

^1^Data for 0 cycles of freeze/thaw is from analysis for sample aliquot stored at 2-8°C since the time of collection and analyzed on Study Day 1 (never frozen).

**eTable10**. Limit of Detection Evaluation Hit Rates (# hits/# total wells) Summary. A hit occurs when the maximum relative fluorescent units (RFU) exceed the threshold of 25,000.

| Dilution | [aSyn Seed] in fg/mL^1^ | Hit Rates | | |
| --- | --- | --- | --- | --- |
|  |  | Native Sample P | Synthetic Sample A | Synthetic Sample B |
| 1.00 | 200.0 | 0.72 | 0.89 | 0.67 |
| 0.30 | 60.0 | 0.33 | 0.61 | 0.78 |
| 0.15 | 30.0 | 0.28 | 0.39 | 0.67 |
| 0.08 | 16.6 | 0.28 | 0.56 | 0.83 |
| 0.01 | 2.0 | 0.22 | 0.22 | 0.56 |
| 0.00 | 0.0 | 0.22 | 0.22 | 0.22 |

^1^ applied to synthetic positive samples only


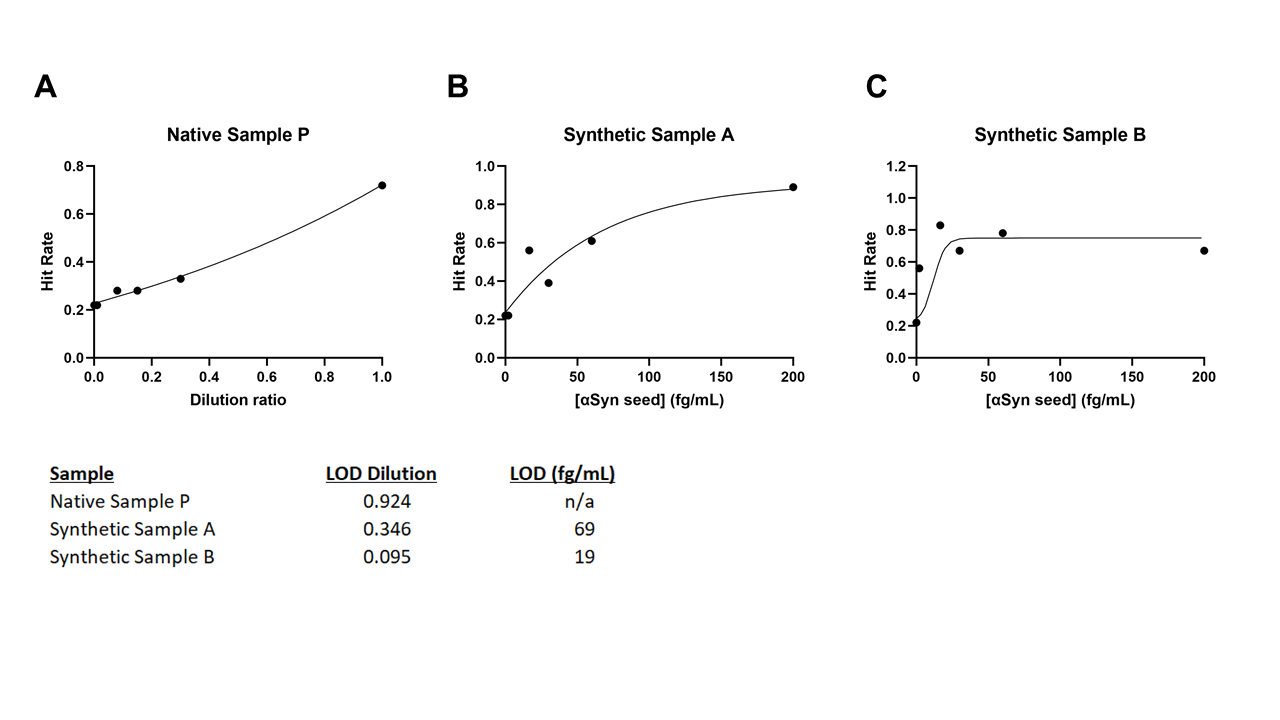
**eFigure1**. Plots of hit rate versus dilution or seed concentration for each of the 3 positive samples are shown. For the synthetic samples, the hit rates as a function of seed concentration are used to estimate the LOD. For this determination, the limit of detection is set to the seed concentration associated with a hit rate of 0.67. For the native PD sample, the dilution ratio that corresponds to the hit rate of 0.67 is determined.
